# Influences on Healthcare-seeking during Final Illnesses of Infants in Under-resourced South African Settings

**DOI:** 10.3329/jhpn.v29i4.8455

**Published:** 2011-08

**Authors:** Alyssa Sharkey, Mickey Chopra, Debra Jackson, Peter J. Winch, Cynthia S Minkovitz

**Affiliations:** ^1^Health Section, United Nations Children's Fund, New York, NY, USA; ^2^Johns Hopkins Bloomberg School of Public Health, Baltimore, MD, USA; ^3^Medical Research Council, Tygerberg, South Africa,; ^4^University of the Western Cape, Bellville, South Africa

**Keywords:** Healthcare, Healthcare-seeking behaviour, Infant mortality, Medicine, Traditional, Qualitative research, South Africa

## Abstract

To examine how health caregivers in under-resourced South African settings select from among the healthcare alternatives available to them during the final illness of their infants, qualitative interviews were conducted with 39 caregivers of deceased infants in a rural community and an urban township. Nineteen local health providers and community leaders were also interviewed to ascertain opinions about local healthcare and other factors impacting healthcare-seeking choices. The framework analysis method guided qualitative analysis of data. Limited autonomy of caregivers in decision-making, lack of awareness of infant danger-signs, and identification of an externalizing cause of illness were important influences on healthcare-seeking during illnesses of infant**s** in these settings. Health system factors relating to the performance of health workers and the accessibility and availability of services also influenced healthcare-seeking decisions. Although South African public-health services are free, the findings showed that poor families faced other financial constraints that impacted their access to healthcare. Often there was not one factor but a combination of factors occurring either concurrently or sequentially that determined whether, when, and from where outside healthcare was sought during final illnesses of infants. In addition to reducing health system barriers to healthcare, initiatives to improve timely and appropriate healthcare-seeking for sick infants must take into consideration ways to mitigate contextual problems, such as limited autonomy of caregivers in decision-making**,** and reconcile local explanatory models of childhood illnesses that may not encourage healthcare-seeking at allopathic services.

## INTRODUCTION

Attainment of the Millennium Development Goal (MDG) 4, i.e. reducing under-five mortality by two-thirds by 2015, requires improvements in the quality of care provided to children and in healthcare-seeking behaviours of their families. A better understanding of the factors that influence healthcare-seeking is critical to ensure that policies and programmes effectively address the constraints families face and build upon the enabling factors that promote appropriate healthcare-seeking.

Studies in other African settings have identified various influences on healthcare-seeking for young children. These include poverty (1), distance to facilities (2), previous negative experiences of mothers with treatment (3), unequal household gender relations (3-5), and women's control of household expenditure (6). Mothers’ explanatory models of illness also impact whether or not and from where they seek treatment (7,8) as do assessments of severity of illness (5,8).

Researchers, using ethnographic methods, emphasize that these processes are locally and culturally specific (9,10). In this study, we examined the factors that influenced healthcare-seeking during fatal illnesses of infants in two under-resourced South African settings.

## MATERIALS AND METHODS

### Design and setting

We conducted qualitative interviews in two sites (Umzimkhulu and Umlazi) with caregivers who experienced an infant death and key informants knowledgeable about health issues relating to women and children. Umzimkhulu is a rural community [population density: 23 per sq km (11)] located in the former Transkei ‘homeland’ with an infant mortality rate (IMR) of 99 per 1,000 livebirths (12). Umzimkhulu has 14 fixed clinics, two district hospitals that provide generalist services to inpatients and outpatients, one specialist hospital, and two mobile clinics (13). Umlazi is an urban township [population density: over 6,000 per sq km (14)] located near Durban with an estimated IMR of 60 per 1,000 livebirths (12). Umlazi has 17 fixed clinics and one regional hospital (15).

### Study sample

In partnership with an ongoing randomized pragmatic trial (‘Good Start’) in the study sites (16), we retrospectively interviewed caregivers who experienced an infant death during the preceding year. Good Start researchers recruited these caregivers between September 2005 and December 2007, when they were at least seven months pregnant or within one week of giving birth.

Routine visits to home**s** took place throughout the child's infancy. When we learned of the death of an infant, we invited the primary caregiver to participate in an interview regarding healthcare and the last illness of the child. Several eligible participants (8 in Umzimkhulu and 7 in Umlazi) were excluded because they could not be located, they had died, and there was no one else appropriate to interview (3 in Umlazi) or they refused participation (1 in Umlazi). We also excluded women who reported that their infants died shortly after birth in the hospital but before discharge (7 in Umzimkhulu and 4 in Umlazi). Our final sample of caregivers included 15 in Umzimkhulu and 24 in Umlazi (Table 1). All but two caregivers were mothers of infants.

We used criterion-based sampling to identify key informants (community leaders and healthcare providers). Eleven key informants interviewed in Umzimkhulu included two community health workers, two traditional healers, one village chief, two village headmen, two community members, and two public-sector nurses (1 hospital-based and 1 clinic-based). Eight informants interviewed in Umlazi included three traditional healers, two local government officials, and three public-sector nurses (2 hospital-based and 1 clinic-based).

### Collection of data

We interviewed caregivers during December 2006–November 2007 using a pretested instrument. After providing background information, the caregivers described their pregnancy and antenatal care (mothers only), labour and delivery care (mothers only), and illness of the infant that led to death (all caregivers). Each participant then responded to a series of semi-structured questions to ascertain the reasons she did or did not seek certain types of healthcare during the last illness of the child. We conducted interviews in the preferred local language (Xhosa or Zulu) of the participants; bilingual field researchers provided translation.

**Table 1.** Characteristics of caregivers and infants

Characteristics	Umzimkhulu (n=15)	Umlazi (n=24)
Type of caregiver		
Mother	14	23
Grandmother	1	1
Age (years) of caregiver (range)		
Mother	17-36	17-36
Grandmother	44	53
Unknown	3	5
Maternal parity		
1	9	14
2-4	5	9
≥5	1	1
Education of caregiver		
None	0	0
Primary	3	3
Secondary or more	8	14
Unknown	4	7
Maternal HIV status at recruitment (obtained verbally)		
Known positive	4	11
Unknown/negative	11	13
Maternal antenatal care (only asked of maternal participants)		
None	0	0
1-3	11	5
4+	1	15
Unknown	3	4
Skilled attendant at birth (only asked of maternal participants)		
Yes	11	22
No	3	1
Unknown	1	1
Sex of infant		
Male	5	12
Female	10	12
Age (days) of infant at death		
<1	3	1
1-28	2	0
29-365	10	23

We also conducted key-informant in-depth interviews in March 2007 to document their assessments of the factors influencing healthcare-seeking practices. Although most of these interviews were carried out in English, in some cases, a bilingual field researcher assisted with translation.

### Analysis of data

Framework analysis, a policy research-oriented analytic approach, guided qualitative analysis of data (17). We indexed transcript data from both caregivers and informants to develop a matrix of the constraints and enabling factors cited as influencing healthcare-seeking decisions. We then conducted community meetings and triangulated data via key-informant interviews to verify information. In addition to exploring *a priori* concepts, we explored emergent themes and patterns in subsequent interviews.

We grouped factors influencing healthcare-seeking into contextual factors, health system factors, and caregivers’ explanatory models of child illness. Contextual factors represent those aspects of a caregiver's household, community or personal situation that influence their living conditions, resources, and opportunities. Health system factors refer to caregivers’ assessments of performance of health workers and accessibility, including physical access, financial access, and availability of services (18). Explanatory models of child illness, as adapted from Garro (19) and Kleinman (20), represent specific interpretations of caregivers about symptoms of their infants. These explanatory models can include pathogenic agents or events originating from outside the body, such as ‘witchcraft’ or ‘evil spirits’, what Young termed ‘externalizing causes’ (21).

We entered, cleaned, and managed qualitative data using the NVivo software (version 7.0). A local Study Advisory Group provided critical assessments of analytic themes and conclusions.

### Ethical approval

The Institutional Review Board of the Johns Hopkins Bloomberg School of Public Health and the Research, Ethics and Study Leave Committee at the University of the Western Cape, South Africa, provided ethical approval. Researchers read translated consent forms, including information about the study aims, potential risks and benefits, compensation, and contact information to prospective participants and provided a signed copy.

## RESULTS

The caregivers used at least one of five types of healthcare during the last illnesses of infants (public health services, general practitioners, traditional healers, home care, or no care), and many caregivers relied upon more than one type (Table 2).

**Table 2.** Types of care provided to infants during final illness

Type of care	Umzimkhulu (n=15)	Umlazi (n=24)
Public (government) health services		
Clinic	0	10
Clinic as a first point of care	0	7
Hospital	8	21
Hospital as a first point of care	0	7
General practitioner (private sector, independently operating medical doctors)	5	6
General practitioner as a first point of care	2	4
Traditional healer	5	4
Sangoma (diviner)	2	2
Inyanga (herbalist)	1	0
Divine healer (faith/ spiritual healer)	2	2
Traditional healer (any type) as a first point of care	1	0
Home care (over-the-counter medications and/or traditional home remedies)	12	15
Home care as a first point of care	10	6
No care	2	0

### Contextual factors

#### Limited autonomy in decision-making

The most important contextual factor associated with healthcare-seeking was the limited autonomy of women in decision-making. In both the sites, most caregivers (12 of 15 in Umzimkhulu and 15 of 24 in Umlazi) reported that they relied on advice from others or felt that they needed to consult others about what to do during final illness of their children. For example, one caregiver in Umzimkhulu reported that when she became aware of danger-signs that indicated that the baby needed treatment, ‘I was alone at home and could not make the decision on my own to take the baby to hospital.’ Her baby died at home the same day. The experience of the caregiver of Umzimkhulu suggests that local policies can perpetuate limited autonomy of the caregivers. This mother reported that, during her pregnancy, clinic nurses told her that her baby's heartbeat was abnormal and that she should see a doctor in the hospital. However, instead of referring her straight to the hospital, “these nurses told me that they were not allowed to call for an ambulance before my family members had been informed. So, I did not go to hospital straight away as I needed to report at home first. At home, my husband and elders refused me to go to hospital… they told me that they would first use traditional muti on me.”

Some caregivers reported feeling conflicted about following the advice of others. For example, when an Umlazi mother noticed that her baby was ‘floppy’ and ‘refusing feeds’, she wanted to go to the hospital but her own mother and another relative said that there was ‘no need because the baby is teething’. Eventually, this baby was taken to a hospital but died while waiting in the queue. Another Umlazi mother whose ‘weak and floppy’ two-month-old child died after treatment from a divine healer said, “I listened to my elders who advised me to send her to a traditional healer rather than to a hospital. They wanted to find out who was behind her illness. They thought that she is [sic] bewitched. If I had taken her to a hospital, she would be alive today.”

Among the informants, there was disagreement about the extent to which family members influence how a mother cares for and seeks treatment for her child (Box 1).

### Health of caregivers

Another contextual factor identified but of less importance was caregivers’ own personal health. Two caregivers (1 in Umzimkhulu and 1 in Umlazi) stated that their own poor health prevented them from taking their sick infants for timely treatment. The Umzimkhulu caregiver, hospitalized herself when her child became sick, said, “I just wish I was at home when my baby got ill because I could have recognized the problem early and sought medical advice.”

**Box 1. Informant's views on the influence of family members on caregiver treatment decisions**

“If the mother-in-law believes that the child should be taken to the witch doctor or the traditional healer, the daughter-in-law then has no say on that. She has to do what the mother-in-law says. Most of the time, the pressure is from the in-laws. And then they are doing what they can to respect the in-laws. Because it is their duty to respect.” (a Community Health Worker in Umzimkhulu)

‘It is very rare that the [child's] mother would not make the decision regarding healthcare…. Even those who do stay with the in-laws, they have got no problem.” (a Headwoman in Umzimkhulu)

### Health system factors

The most important health system factors influencing healthcare-seeking were physical access, financial access, availability of services, and performance of health worker.

#### Physical access

Most caregivers (13 of 15 in Umzimkhulu and 18 of 24 in Umlazi) cited physical access to facilities as an important influence on whether and when they sought care. In Umzimkhulu, all the caregivers who eventually took their children to the hospital said that they delayed going there and first provided home care due to distance. Distance and difficulties in finding transport were cited as the primary reasons. Another Umzimkhulu caregiver only provided home care to before death of her child. In both the sites, most (22 of 29) caregivers who sought hospital care used a public bus, a taxi, or a private (hired) car to get there. In Umzimkhulu, two caregivers each called for an ambulance when the child was sick; neither of which arrived. One of these women said that she eventually hired a private car, and the other stayed at home with her child where he died the next morning. In Umlazi, only one caregiver called for an ambulance for her child which arrived promptly. Four caregivers (2 in Umzimkhulu and 2 in Umlazi) who chose other forms of transport said that they did so after having waited several hours for an ambulance to arrive on previous occasions.

Private transport services were also problematic. Two women (Umzimkhulu) each reported that it took more than six hours for a hired private car to arrive at their home. The private car a third caregiver (Umzimkhulu) hired also arrived several hours after her baby died.

In Umlazi, two caregivers were not able to take their babies for care immediately because it was at night, and they did not feel safe travelling.

#### Financial access

An inability to pay for transport was a related constraint (5 in Umzimkhulu and 6 in Umlazi). One Umlazi caregiver whose child became ill at night reported that getting an ambulance at that time was ‘problematic’, and she could not afford to hire a private car. She waited until morning when a more affordable form of transport was available and then went to the closest facility (a clinic). She said, “if I had taken the baby directly to the hospital, he would still be alive.” Another mother in Umlazi reported that her newborn baby began breathing fast on the bus ride while returning home after discharge from the hospital. She wanted to return directly to the hospital but did not have enough money for transport. The baby died as soon as the mother arrived at her bus stop.

#### Availability of services

Lack of availability of service also influenced caregivers (6 in Umzimkhulu and 6 in Umlazi). In Umlazi, for example, one mother was turned away by the clinic because it had ‘filled’ its daily quota. Three mothers in Umlazi incorrectly believed that they could not take their very sick infants to the hospital without a referral letter from a clinic. Two caregivers in Umlazi described their difficulties in accessing services because a government strike was underway during final illness of their children. One developed chest indrawing, apnoea, and seizures a week after the discharge from the hospital. Since the hospital was affected by the strike, the caregiver consulted a local general practitioner (GP) who referred the mother to a semi-private hospital which is located outside the area. As ambulance drivers also were on strike, the mother took two different taxis in an effort to reach the hospital but her baby died along the way.

Assessments of limited hours of operation at public facilities, insufficient staff and medicines, and long-waiting times also influenced the caregivers (6 in Umzimkhulu and 6 in Umlazi). One Umzimkhulu mother, who despite the financial and transport difficulties, took her child to a GP two hours away, stated, “I do not like using the clinic because most of the time there is no doctor visiting the clinic or sometimes there might not be a sister [nurse] to examine the child. So, I prefer to use my doctor because I have used him for many years, I trust him, and I do not mind paying R 50 to get there.” One Umlazi caregiver who waited a day before taking her infant for care said, “The clinic only sees antenatal cases on the day I wanted to take my son there.”

The informants also mentioned some of the access problems cited by caregivers (Box 2).

**Box 2. Informant's views on access to care**

“Sometimes people are complaining that they take a long time to be attended [to]. Sometimes a person comes, they take about 3, 4, and 5 hours waiting. If people come for sickness, they are suffering, they are in severe pain. They must quickly get attended. The people will go to hospital and say [to me], “They don't take care of us. We just lie like that, and they are moving up and down without seeing us while we are in severe pains.” (Inyanga in Umlazi)

#### Performance of health workers

Ten caregivers in Umzimkhulu and 13 in Umlazi reported being influenced by their own previous experiences with health workers and/or the experiences of others. Provider demeanour was important, as was how successful the caregiver viewed previous treatments. One Umzimkhulu mother who reported having had hospital nurses shout at her when she delivered her baby, chose to take the child first to a traditional healer when he became ill. Her baby died just after they arrived back home from the healer. However, she still rated his care as ‘good’ because “he showed that he cares about people and my baby was helped immediately.”

The informants also commented that the provider's attitudes can deter healthcare-seeking (Box 3).

#### Caregivers’ explanatory models of child illness

The caregivers’ explanatory models of their children's illness also influenced healthcare-seeking. Of particular importance were assessments of the caregivers regarding the severity of illness and danger-signs of their children and their attribution of the illness to an externalizing cause, e.g. ‘evil spirit’.

**Box 3. Informant's views on provider attributes that influence healthcare-seeking**

“It's the treatment from nurses that could stop [a mother with a sick child] from going to health services. They get scolded. If the woman does have money, she would rather prefer going to private doctors. If she doesn't have money, some may rather stay at home.” (a Headman in Umzimkhulu)

‘To be honest, we are to be blamed also as health workers because we do have some barriers that we create. Because, if the client was not looked after well, or there is something you said that she didn't like, she cannot come to you. And if she is coming from the traditional healer and you say, “You decided to come so late with a sick baby! Where have you been?” If you start with those things, it puts her off. She won't be interested in coming back to the institution. There are attitude problems.” (a hospital nurse in Umzimkhulu)

#### Assessment of severity of child's illness/infant danger-signs

Several caregivers (6 in Umzimkhulu and 13 in Umlazi) reported that they did not realize the gravity of symptoms of their babies and, therefore, delayed seeking treatment. One Umlazi mother said that she stayed home with her child for a week before taking her for treatment because “I did not think it was serious. I thought that it was due to cold weather and it will subside.” Other caregivers reported thinking that problems, such as ‘floppiness’, ‘refusing feeds’, and diarrhoea, were normal problems associated with teething and that treatment was unnecessary.

One informant (Headwoman in Umzimkhulu) suggested that the perceived severity of illness determines whether caregivers go to a traditional healer or to western medical services: “When the baby is very ill, we prefer the western healers like the clinic—not the traditionals. People use the traditional healers but not for a very sick person.” However, no caregivers reported this practice. Instead, several caregivers suggested that it is appropriate to consult western providers or traditional healers interchangeably depending on which treatments seem to be working.

#### Attribution of illness to an externalizing cause

Both caregivers and informants reported that, if the cause of illness is understood to be witchcraft or angry ancestors, families prefer to consult traditional healers (Box 4). Six caregivers (3 in Umzimkhulu and 3 in Umlazi), while concurrently mentioning danger-signs, such as diarrhoea, vomiting, visible pain, and fast breathing, attributed the illness of their children to an externalizing cause that required traditional treatment. One Umzimkhulu caregiver whose baby had diarrhoea and vomiting for two days said, “a red mark at the back of the [baby's] head was a danger-sign showing that the baby was not well.” She took her baby to a traditional healer ‘who was good in healing the mark on babies.’ However, since it was at night when they visited the healer, they were told to come back the next day. Instead, she took her baby to the hospital where she died 12 hours later. She said that she wished that the baby could have received care from the healer, stating, “If I could have another baby, I would make sure that if I notice that red mark on the baby's head I quickly run for help.” Another caregiver in Umlazi reported that her baby had pain, dehydration, and rapid breathing for three days and was eventually admitted for pneumonia to the local hospital. She said that her baby had *‘Iplayit’*, meaning ‘the baby cries a lot and has sunken fontanelle’ and that to treat the baby, “We burned herbs for her to inhale and informed our ancestors.”

**Box 4. Informantt's views on caregiver explanatory models that influence care-seeking**

“Some of them, they have got a belief that you don't just get sick because there is a bacterium or virus that you have contacted. You have been bewitched. That's what make[s] them get treatment from traditional healers. Because they say traditional healer is going to give them medicine that is going to prevent this evil spirit that comes from their neighbours to bewitch them. So, the child will be the last one to be taken to the hospital.” (a Hospital Matron in Umlazi)

## DISCUSSION

This study aimed to elucidate the factors influencing healthcare-seeking during final illnesses of South African infants. Experiences of women highlight the complexity of intra-household relationships and treatment decision-making dynamics. Although all the participants self-identified as the primary caregivers, most (27 of 39) caregivers relied on advice or consulted others regarding what to do during the final illness of their children. Health workers may reinforce women's feelings of limited autonomy. Indeed, it has been reported that the relationship between clients and health workers in South Africa is characterized by power and hierarchy (22). In addition, gender inequality and low social status have been found to influence the health of South African women (23). The results of the present study suggest that interpersonal power dynamics may affect the health of their children as well.

Although public health services in South Africa are free, poor families still face financial constraints that limit access to healthcare. In other African settings with minimal direct costs, indirect costs, e.g. transport or costs for childcare, have been found to influence healthcare-seeking (24). Similar to these findings, problems of physical access and unreliable/unavailable transport have been reported as a problem in other low-resource settings in South Africa (25).

Maternal dissatisfaction with quality of healthcare in South African public facilities has been reported, particularly due to long-waiting and short consultation times and lack of access to doctors (26,27). In this study, the caregivers reported that negative attitudes of the providers led them to seek healthcare elsewhere. Further, many South African allopathic providers do not approve of other types of healing (28). Consequently, a caregiver may be unwilling to seek healthcare from a provider who disagrees with her explanatory model of the illness, or she may be unwilling to provide a full history of the illness and treatments given when she does present (29). The failure to fully disclose treatment history can have important implications for the health outcomes, particularly with respect to dangerous interactions of allopathic and traditional medicines (29). Finally, each of the six caregivers who attributed the deaths of their babies to an externalizing cause also reported symptoms that should have been recognized as danger-signs. As has been reported in other African settings (30,31), poor healthcare-seeking can be related to lack of recognition of diarrhoea, coughing, and fast breathing as important danger-signs.

Finally, although the characteristics of the caregivers in both the sites were similar in many respects (Table 1)., limited autonomy and long distances to facilities appeared to be more pronounced in Umzimkhulu. In addition, while transport problems were reported in both the sites, safety concerns relating to access at night were reported only in Umlazi. In other respects, the influences on healthcare-seeking reported by the rural participants were surprisingly similar to those reported by the urban participants.

### Implications for programmes and policies

Policy and programmatic initiatives to improve timely and appropriate healthcare must be multifaceted, taking into consideration how to improve the use of health services, e.g. by addressing inadequacies in access and performance of health workers and by improving knowledge of infant danger-signs that require care, and determining whether, and how, the health system can better compensate for exogenous factors, such as limited decision-making autonomy and financial difficulties of caregivers and local explanatory models of childhood illnesses. This is particularly important as many of the caregivers reported more than one influence on their decisions to seek healthcare. The myriad of social and systemic dynamics in which healthcare-seeking processes are embedded must be important considerations in the design and implementation of interventions.

This study demonstrated a need to improve education on infant danger-signs at each antenatal and paediatric contact. Interventions that involve other family members or that disseminate behaviour-change messages to the broader community may further promote these messages, particularly in settings where autonomy of women in decision-making is limited (3,32). These messages should be sensitive to local explanatory models of illness causation and cultural practices (7). Public health staff also should be sensitized, particularly given that many have a negative view towards traditional healing and healers (33). However, a patient-centred approach that “accommodate[s] diverse patient needs and preferences” (34) will promote collaboration among all types of providers in these settings.

### Limitations

Data on healthcare-seeking were based on recollection of caregivers and may be subject to error. To reduce this problem, the interviewers confirmed the statements of participants with follow-up questions to identify the discrepancies or omitted information and correct inconsistencies.

In addition, the professional backgrounds of the interviewers may have evoked desirable answers, e.g. an under-reporting of treatment with traditional medicines and by healers, although efforts were made to minimize this by providing assurances to the participants during the informed consent process that there were no right or wrong answers, that their comments would be anonymous and confidential, and that the interview was not in any way intended to be judgmental.

### Conclusions

There is considerable inter-connectedness between the various MDGs, including eradicating poverty, reducing child mortality, and promoting gender equality. The findings of the present study indicate some ways that poverty, limited autonomy in decision-making, poor access to and quality of healthcare, and local understandings of illnesses combine to result in high rates of infant death. Initiatives that address the complex interactions among caregivers, the health system, and the broader social, economic and cultural context in which families live are likely to be more effective, balanced, and sustainable in reducing infant deaths.

## ACKNOWLEDGEMENTS

This research was funded by the Eunice Kennedy Shriver National Institute of Child Health and Development, Rockville, MD (R03HD052638). The authors thank the health providers, community leaders, and, particularly, the caregivers who generously shared their thoughts and experiences to inform the study. They also thank the Good Start research team who facilitated and contributed to the study. The content is solely the responsibility of the authors and does not necessarily represent the official views of the National Institute of Child Health and Development or the National Institutes of Health.
